# Pulp Vitality Testing with a Developed Universal Pulse Oximeter Probe Holder

**DOI:** 10.3390/medicina57020101

**Published:** 2021-01-23

**Authors:** Živilė Grabliauskienė, Roberta Zamaliauskienė, Greta Lodienė

**Affiliations:** Department of Dental and Oral Pathology, Lithuanian University of Health Science, Eiveniu 2, LT-50161 Kaunas, Lithuania; grab.zivile@gmail.com (Ž.G.); lodieneg@gmail.com (G.L.)

**Keywords:** pulse oximeter, pulp vitality tests, CAD, clinical outcomes, clinical practice guidelines, clinical studies/trials, diagnostic systems, endodontics

## Abstract

*Background and Objectives* An accurate determination of the pulp status is relevant for a proper endodontic diagnosis. Objectives: The aim was to develop a universal pulse oximeter probe holder for measuring the oxygen saturation and to evaluate the use of pulse oximetry as a test for pulp vitality, by comparing the levels of oxygen saturation in the index finger and in the healthy dental pulp. *Materials and Methods* The universal holder was designed with software and printed with a 3D printer. The study was carried out on 128 healthy teeth. They were divided into eight groups according to tooth type. Ten root canal treated teeth served as a negative control group. For each patient, a pulse oximeter was first applied on the tooth followed by the index finger. The significance level (α) was set at 0.05. *Results:* The developed and manufactured universal pulse oximeter probe holder was suitable to measure the pulp vitality of all types of teeth. The handle allowed for holding the pulse oximeter on the tooth in parallel, firmly and securely. Significantly higher oxygen saturation was observed in the index finger (97.22%) compared to the dental pulp (93.17%) (*p* < 0.001). No correlation was observed between the maxillary teeth and index finger oxygen saturation values (*r* = 0.05, *p* = 0.72), whereas, between the mandibular teeth and index finger, a positive correlation was detected (*r* = 0.29, *p* = 0.02). There were no significant differences in the pulp oxygen saturation values between different teeth groups. *Conclusion:* The newly developed universal pulse oximeter probe holder is an effective device for pulp vitality testing.

## 1. Introduction

The purpose of diagnosis in endodontics is to determine the condition of a painful tooth and to identify the cause of the pain or discomfort. Scientific knowledge in this field is mandatory along with available clinical diagnostic devices [1].

Electric pulp and thermal tests are widely used diagnostic tests related to the neurophysiology of the dental pulp. The electric pulp tester is designed to deliver an electric current to stimulate vital nerve fibers and measure the neural transmission; however, it does not indicate the health or integrity of the pulp [2]. Additionally, the device gives a false negative response to recently traumatized teeth with intact vascularity and lost sensitivity [3,4] and gives a false positive response to teeth that are partly necrotic and lack a blood supply [5].

During thermal tests, cold or heat is applied to the tooth to stimulate the nerve fibers to produce pain [6]. However, the vitality of the pulp is determined by the state of the vascular supply, whereas these pulp tests determine the neural response [2]. The pulp tissue might not necessarily be innervated but still have a sufficient blood supply [7]. Accordingly, the research is intended to objectively measure the vitality of the tooth while performing a non-invasive procedure [8].

The laser Doppler flowmetry (LDF) technique was first described in 1986 by Gazelius et al. based on the electro-optical technique [9]. Laser light travels to the pulp using dentinal tubules as guides [10,11]. In spite of the many advantages of LDF (accurate, reliable, reproducible, non-painful, useful in luxation injuries, and for young children), this method has limitations (too expensive for use in a dental office, the sensor should be kept motionless and in constant contact with the tooth for accurate readings, and the laser beam must interact with the moving cells within the pulpal vasculature) [12,13].

Contamination caused by backscattered light from the periodontal tissues [14] cannot be completely eliminated and gives false-positive results [9,15]. Blood pigments within a discolored tooth crown can also interfere with laser light transmission [16]. These disadvantages make LDF difficult to use in everyday clinical practice.

Pulse oximetry is a completely objective test to evaluate vascularization by determining oxygen saturation level in the circulating arterial blood [16]. The pulse oximeter probe emits a red and an infrared light to the pulp. Both types of light are needed because oxygenated and deoxygenated hemoglobin are different in color and absorb different amounts of red and infrared light. The pulsatile blood circulation formed absorbance peaks, which were measured using the photodetector. The pulse rate and oxygen saturation level were interpreted from measured data [17,18]. No input from the patient is required; therefore, pulse oximetry is a completely objective test to evaluate vascularization [16]. Unfortunately, there are currently no devices adapted for dental use. In recent studies, pulse oximeter probe holders were created but designed only for a particular group of teeth. However, the authors achieved reliable results and concluded that pulse oximetry can be used in practice as an accurate vitality test [19,20,21].

The aim of this study was to develop a universal pulse oximeter probe holder for measuring oxygen saturation and evaluate the use of pulse oximetry as a test for pulp vitality, by comparing the levels of oxygen saturation in the index finger and in healthy dental pulps.

## 2. Materials and Methods

The study was performed in the Department of Dental and Oral Pathology, Lithuanian University of Health Sciences, during the period extending from September 2017 to January 2019. The study was approved by the Lithuanian University of Health Sciences Ethics Committee.

Patients who met the inclusion criteria were invited to participate in the study. The inclusion criteria were as follows: the teeth free of caries, fractures, and discoloration; good oral hygiene; no bleeding while probing; periodontal probing depth between 1 mm and 3 mm; no tooth mobility; normal response to the cold test; no history of pain or trauma; no pulp chamber calcification; no periapical changes, internal or external resorption; and no pain on percussion and palpation. Patients with root canal-treated teeth were chosen for the negative control group. Patients with orthodontic braces, prosthetic crowns, periodontal probing depth more than 3 mm, and periapical changes in radiographs were excluded. The patients did not have a systemic disease and did not consume drugs.

A total of 138 patients were recruited for the study, and the patients received oral and written information about the study and signed an informed consent form. They had the right to decline participation in the study at any time.

### 2.1. Development of the Universal Pulse Oximeter Probe Holder

**Figure 1:**
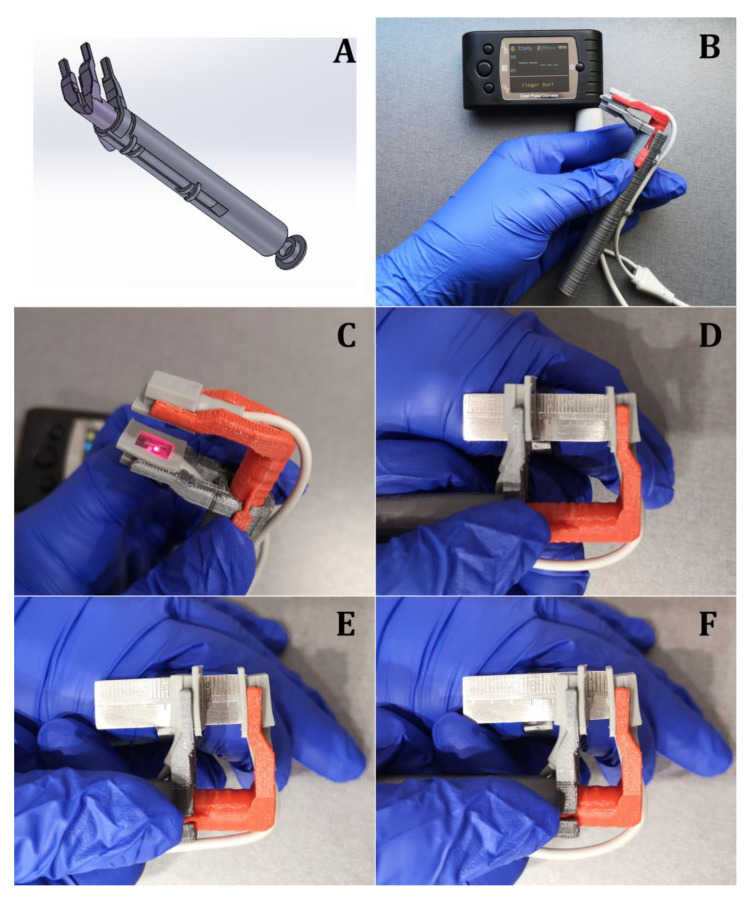
(**A**)—3D CAD (computer-aided design) model. (**B**)—Designed pulse oximeter holder. (**C**–**F**)—Sliding calipers principle.

According to the anatomy and measurements (mean width and height and position of the pulp chamber in every type of tooth) of the teeth, a 3D CAD (Computer-aided design) model of the holder was designed using the Solidworks program (Figure 1A) and printed with a Replicator 3D printer (MakerBot Industries, New York, NY, USA). The universal pulse oximeter holder was made from PLA (Polyactic acid) plastic to enable disinfection (Figure 1B).

The universal pulse oximeter holder was designed based on the sliding calipers principle and ensures parallel placement of the diodes. Thus, it can be used for all widths of teeth while maintaining parallelism and provide accurate measurements (Figure 1C–F).

After medical and special anamnesis of the patient, periapical radiographs were taken before the pulp testing procedures.

A total of 128 healthy asymptomatic teeth were divided into eight groups according to tooth type (incisors, canines, premolars, and molars from both jaws), with 16 teeth in each group. Ten root canal treated teeth with no abnormality detected in radiographs and with no response to thermal tests served as a negative control group.

A cold test was performed using ethyl chloride spray (Polydent, Lodz, Poland). The teeth were isolated and dried with cotton rolls, and an ice spray was used with small cotton pellets and pincers. These pellets were placed on the middle third of the buccal surface of the teeth. If no pain occurred within 15 s, the tooth pulp was defined as necrotic and was removed from the study.

The tooth surfaces were dried and isolated with cotton rolls. The oxygen saturation levels of all teeth were recorded with a pulse oximeter CMS60C (CONTEC, Qinhuangdao, Hebei Province, China). The pulse oximeter probe was placed on the tooth, light-emitting diodes (LEDs) were placed on the labial or buccal surface, and the light detector was placed on the lingual or palatal surface. The LEDs and photodetector were parallel. The LEDs were placed on the gingival third of the tooth, avoiding the gingiva. Patients were instructed to keep their head still so as not to influence the readings. After measurements became stable, the readings of oxygen saturation in the pulp were recorded. Measurements were repeated three times, and the mean of the values was calculated.

**Figure 2:**
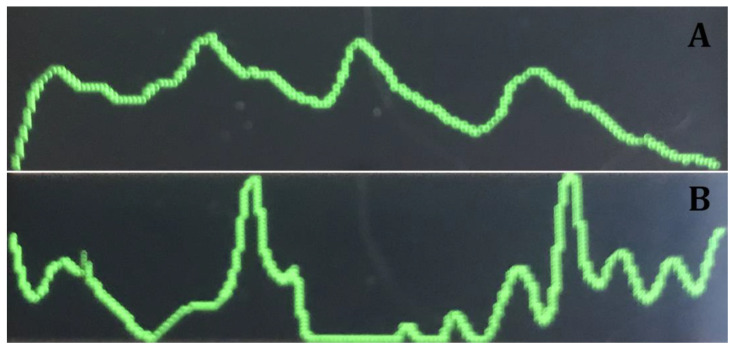
(**A**)—Graph when measuring oxygen saturation of the dental pulp. (**B**)—Graph when measuring oxygen saturation of the gingiva.

The oxygen saturation of the index finger was measured for each patient. It was advised to measure pulp oxygen saturation in the middle third of the tooth crown [12]; however, in older patients, the pulp chamber is retracted, and measuring in the center of the tooth crown may distort the results. Therefore, LEDs of the pulse oximeter probe must be placed on the gingival third of the tooth but avoiding the gingiva to prevent interference from the gingival oxygen levels [19]. The silicone surrounding LEDs provides a socket to fit the tooth equator. Additionally, we observed that, when the device measured the oxygen saturation in the tooth versus in the gingiva, the curves on the pulse oximeter’s display were different (Figure 2A,B).

### 2.2. Statistical Analysis

The data were collected and statistically analyzed using the Statistical Package for Social Sciences program (SPSS) 21.0 (Chicago, Illinois, IL, USA). A *p*-value of <0.05 was considered statistically significant. The quantitative variables were described as arithmetic mean (standard deviation (SD)) and non-normal variables were reported as median (interquartile range (IQR)). Continuous variable normality assumption was verified using the Kolmogorov–Smirnov test. Test of the normality of the investigated variables was denied—the distribution was not normal; therefore, the non-parametric Mann-Whitney and Wilcoxon tests were used. Cases with more than 2 groups were compared using Kruskal-Wallis test. The correlation coefficient was calculated using the Spearman test.

## 3. Results

**Figure 3:**
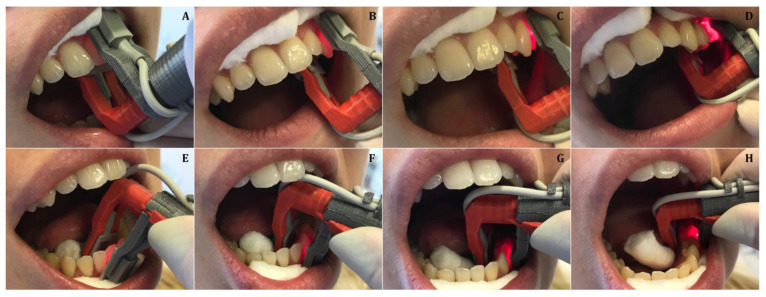
(**A**–**H**)—Placement of the pulse oximeter probe on different types of teeth.

The developed and manufactured universal pulse oximeter probe holder was suitable to measure the pulp vitality of all types of teeth. The handle allowed for holding the pulse oximeter on the tooth in parallel, firmly and securely (Figure 3A–H).

The age of the patients ranged from 20 to 54 (mean age was 26.0 years (SD 6.7) (median (25–75%) 24.0 (22.25–26.0))) in this study and had no correlation with oxygen saturation in teeth (*r* = 0.01; *p* > 0.05) or in the index finger (*r* = −0.08, *p* > 0.05). In all patients, the oxygen saturation level in the index finger was significantly higher than the oxygen saturation in all types of teeth (*p* < 0.001): 97.22% (98.0 (96.0–99.0)) and 93.17% (94.0 (89.25–97.0)), respectively. There were no significant statistical correlations between the blood oxygen levels in the index finger and in the maxillary teeth (*r* = 0.05, *p* = 0.72), although the mandibular teeth showed a positive correlation (*r* = 0.29, *p* = 0.02). No statistically significant differences were found in the oxygen saturation values between all types of teeth (*x*^2^ = 2.24, *df* = 3, *p* = 0.53), as well as when the teeth of both jaws were compared (*p* = 0.23) (Table 1). The control group (10 non-vital teeth) showed 0% oxygen saturation with a statistically significant difference from the healthy teeth (*p* < 0.05).

The results were obtained: the oxygen saturation level in teeth—93.17% (4.87) and in the index finger—97.22% (1.84), *r* = 0.066, with type I error 0.05, statistical power was >0.9—considered as significant.

## 4. Discussion

The pulse oximeter probe holder developed and manufactured in our study differs from others, as the device was presented with a newly developed universal probe holder that conforms to the size, shape, and anatomy of the tooth and can be used on all types of teeth. The LEDs and photodetector are in parallel to each other and can be held firmly. This increases the accuracy of measurements, which is very important for the objective evaluation of teeth vitality in daily clinical practice [19]. In previous studies, authors attempted to adapt or make probe holders, but they were adapted only to a particular type of teeth. Mainly stainless-steel clips and rubber dam clamps were used as the base for the holders.

Noblett et al. [17] modified a rubber dam clamp and attempted to detect the pulpal blood circulation in vitro. Kahan et al. [22] conducted an in vivo study and measured the oxygen saturation level in incisors using the same technique. Goho modified an ear probe to evaluate the pulpal blood oxygen saturation in maxillary incisors [23]. Gopikrishna et al. used plastic materials attached to rubber dam forceps to make a probe holder for the maxillary anterior teeth. However, these methods do not maintain parallel placement of the LEDs and photodetector of the probe [24,25,26]. Stella et al. developed a stainless-steel pulse oximeter probe holder adapted for the maxillary central incisors only [20].

Estrela et al. invented two stainless-steel holders, one for maxillary premolars and the other for molars [21,27]. These holders maintained parallelism; however, they were typically designed to fit one type of tooth, and so there was often unnecessary space between the probe and the tooth, which could distort the results. Even if previously designed pulse oximeter probe holders were introduced to the market, they would be impractical for dentists, and the use of several probe holders would complicate and prolong the work. Our developed probe holder is universal and could be easily replicated using a 3D printer.

In our study, no correlation between the patient’s age and oxygen saturation in the teeth or in the index finger was observed. These results contradict a C. Estrela et al. study, where significantly lower levels of oxygen saturation (80.0%) were found in the oldest age group (40–44) compared to the other groups: 89.71%, 87.67%, 88.71%, and 84.80% for age groups 20–24, 25–29, 30–34, and 35–39 years, respectively [21]. Such a difference may be because, in our study, we did not divide the patients into age groups, and the mean age was 26 years old.

In our study, the oxygen saturation levels of the index finger were higher than in the tested teeth. These findings are in accordance with previous studies (Table 2) [19,21,24,28,29,30]. The explanation of the higher oxygen saturation in the index finger compared to the teeth could be that the diffraction of the red and infrared lights by the tooth enamel and dentin can cause a decrease in the measured values [30].

No correlation between the oxygen saturation in the maxillary teeth and patient index finger was observed in our study. These findings are in accordance with previous studies [20,27,28,29]. However, Sadique et al. found a very weak correlation [30]. That result could be explained by the fact that the oxygen saturation was measured only in the maxillary anterior teeth, while in our study, measurements were taken from all maxillary teeth. Sharma et al. found a strong positive relationship between the tooth and finger oxygen saturation values in the vital permanent teeth [31]. Although the age of the patients in their study was from 4 to 12 years old, no data regarding the root formation stage was specified. Therefore, the results of this study could be inaccurate in the case of including immature teeth.

We found a positive correlation between the oxygen saturation in the mandibular teeth and index finger. This could be due to higher values of oxygen saturation in the lower teeth compared with the maxillary teeth, as C. Estrela et al. found in their study, with a higher mean for the mandibular molars (86.89%) compared to the maxillary molars (83.59%) [27].

No significant differences in the oxygen saturation values between all types of teeth were observed in our study. These results are in agreement with previous studies (Table 2) [24,28,29]. In Estrela et al.’s study, however, there was a significant difference in the mean of the oxygen saturation between the first (85.76%) and second maxillary molars (81.87%). This could be due to the unequal number of teeth in the groups of their study [27].

## 5. Conclusions

This study confirmed the potential clinical use of a pulse oximeter with a manufactured universal holder as a dental pulp vitality tester. However, further in vivo studies must be conducted to evaluate the dental pulp oxygen saturation levels in different pulp stages.

## Figures and Tables

**Table 1:** The oxygen saturation level in all types of teeth.

Jaw	Tooth Type	*N*	Oxygen Saturation (%)		Range (%)
			Mean (SD)	Median (25–75%)	
Maxillary	Incisor	16	93.75 (3.26)	94.5 (91.0–96.0)	88–99
	Canine	16	91.63 (4.98)	91.5 (89.0–95.75)	79–98
	Premolar	16	93.38 (5.76)	96.0 (89.5–97.75)	80–99
	Molar	16	92.31 (4.42)	91.0 (88.25–96.0)	87–99
Mandibular	Incisor	16	93.69 (6.22)	95.5 (91.25–98.75)	75–99
	Canine	16	93.56 (3.6)	94.0 (91.0–96.5)	87–99
	Premolar	16	93.94 (4.74)	96.0 (88.25–98.0)	86–99
	Molar	16	93.13 (5.82)	94.0 (88.25–99.0)	82–99

**Table 2:** The oxygen saturation values of the index finger and tested teeth in previous studies.

	Authors/Year	Index Finger Saturation	Tested Teeth/Saturation (Mean)	Statistically Significant Differences between Tested Teeth
1	B. Anusha et al./2017 [19]	98.4%	Max. and mand. front teeth—94.6%	-
2	Calil et al./2008 [28]	95%	Max. c. incisor—91.29% Max. canine—90.69%	No
3	M.H. Pozzobon et al./2011 [29]	92.85%	Max. front teeth—85.27%	No
4	M. Sadique et al./2013 [30]	95.88%	Max. front teeth—85%	-
5	C. Estrela et al./2017 [27]	92.89%	Max. molars—83.59% Mand molars—86.89%	Yes No
6	C. Estrela et al./2017 [21]	93.7%	Max. premolars—86.2%	-
7	V. Gopi Krishna et al./2006 [24]	97.58%	Max. front teeth—79.59%	No
8	Present study	97.22%	All types of teeth—93.17%	No

## Data Availability

Data are contained within the article.
